# Knockdown of RBM15 inhibits tumor progression and the JAK-STAT signaling pathway in cervical cancer

**DOI:** 10.1186/s12885-023-11163-z

**Published:** 2023-07-20

**Authors:** Chunnian Zhang, Liqin Gu, Juan Xiao, Feng Jin

**Affiliations:** Department of Gynecology, Ganzhou People’s Hospital, No. 16, Meiguan Avenue, Ganzhou City, 341000 Jiangxi Province China

**Keywords:** Cervical cancer, N6-methyladenosine, RBM15, The JAK-STAT signaling pathway

## Abstract

**Background:**

RNA binding motif protein 15 (RBM15), a writer of N6-methyladenosine (m6A) methylation, contributes significantly to the development of various tumors. However, the function of RBM15 in cervical cancer (CC) has not been determined.

**Methods:**

Based on the GSE9750, GSE63514, and m6A datasets, m6A-related differentially expressed genes (DEGs) were screened out. The hub genes were identified by generating a Protein-Protein Interaction (PPI) network. RT-qPCR was conducted to assess the mRNA expression of hub genes. CCK8, scratch wound healing, and transwell assays were utilized to examine the influence of RBM15 on HeLa and SiHa cells. Tumor xenograft models were used to assess the effects of RBM15 on tumorigenesis. A mechanistic analysis of RBM15 in CC tumors was conducted using the GeneCards and Coxpresdb databases, followed by a Kyoto Encyclopedia of Genes and Genomes (KEGG) enrichment analysis, and the pathway-related genes were subsequently validated using Western blotting.

**Results:**

Five DEGs were screened, including WTAP, RBM15, CBLL1, and YTHDC2. Among them, WTAP, RBM15, CBLL1, and YTHDC2 were hub genes and can be used as biomarkers for CC. RBM15 expression was considerably increased, while WTAP, CBLL1, and YTHDC2 were significantly downregulated. Knockdown of RBM15 significantly suppressed the proliferation, invasion, and migration of CC cells and tumorigenesis. Moreover, knockdown of RBM15 significantly reduced the expression levels of proteins related to the JAK-STAT pathway.

**Conclusions:**

Knockdown of RBM15 inhibited the progression of CC cells, which probably by inhibiting the JAK-STAT pathway pathway.

**Supplementary Information:**

The online version contains supplementary material available at 10.1186/s12885-023-11163-z.

## Introduction

Cervical cancer (CC) ranks second in the prevalence of female malignancy [1]. The infection of high-risk human papillomavirus [2], early age at first intercourse [3], and smoking [4] are all risk factors for CC. CC tumorigenesis is a complex process involving transcriptomic, genetic, and epigenetic alterations [5]. The stage at diagnosis is a crucial factor in determining the prognosis of CC, but most patients are at an intermediate to advanced stage [6] accompanied by high recurrence and metastasis [7], making treatment more difficult. Therefore, much more research is required to clarify the molecular mechanisms of CC.

N6-methyladenosine (m6A) is an epigenetic alteration, which is methylated at the N6 position of adenosine [8]. Being the most prevalent RNA modification in mammals, m6A regulates multiple processes in RNA metabolism, including splicing, export, and translation [9]. Several studies have demonstrated that m6A affects the progression of various tumors, including CC. However, research on the function and mechanism of m6A in CC is not been fully expounded. RBM15, the m6A-modified catalytic protein, combines with METTL3, WTAP, and METTL14 to form the m6A methyltransferase complex, which is accountable for the installation of m6A [10]. Previous reports suggest that RBM15 plays a significant role in tumor development. The m6A site modified by RBM15 is recognized by IGF2BP3, enhancing laryngeal squamous cell carcinoma progression [11]. RBM15 accelerates the development of hepatocellular carcinoma in an IGF2BP1-dependent way [12]. Knockdown of RBM15 suppresses the growth and metastasis of colorectal cancer [13]. However, the function of RBM15 in CC has not been elucidated.

The Janus kinase/signal transducer and activator of transcription (JAK/STAT) signaling pathway is widely recognized as a pivotal hub in cellular function, serving as a central node for intercellular communication [14]. The JAK/STAT signaling pathway plays an important role in tumor development. The activation of JAK/STAT signaling leads to the upregulation of various proteins involved in cell proliferation, cell survival, stemness, self-renewal, evasion of immune surveillance mechanisms, and overall tumor progression [15]. Enhancement of the epithelial-mesenchymal transition through the activation of the JAK/STAT3 signaling pathway results in increased tumorigenic and metastatic potential, facilitates the transition of cancer stem cells, and contributes to chemoresistance in cancer [16]. In CC, the JAK/STAT pathway has important correlation with HPV E6/E7 oncoprotein [17]. And the activation of the JAK-STAT pathway by CXCL10 and CXCR3 leads to heightened expression of exosomal PD-L1, which may contribute to HPV evasion of the immune response and subsequent development of cancer [18]. In addition, studies have shown that the JAK/STAT signaling pathway is involved in the progression of multiple tumors by regulating m6A-related genes. The cell cycle regulation in human glioma cells is influenced by YTHDC1-mediated VPS25, which specifically targets the JAK-STAT signaling pathway [19]. ZC3H13 has been identified as a factor influencing the progression of hepatocellular carcinoma, which potentially impacts the JAK-STAT signaling pathway [20]. However, the correlation between the JAK-STAT signaling pathway and m6A has rarely been reported in CC.

Bioinformatics allows for faster and comprehensive insights into disease mechanisms and treatments. For example, over the course of just a year, from 2021 to 2022, the Ren et al. team make a series of significant discoveries in Kidney disease. They identifies a biological signature and predictive model for biopsy-proven acute rejection after kidney transplantation, one o six stemness-associated genes predicting overall survival in renal clear cell carcinoma: AC010973.2, and RNA-binding protein genes and drug candidates associated with prognosis in renal papillary cell carcinoma, as well as also examined the DNA damage and prostate cancer correlation, which are investigated by bioinformatic methods [21–24]. And research on the molecular mechanisms of m6A modification in tumors has been greatly facilitated by the rapid development of bioinformatics. In this study, we selected GSE9750 and GSE63514 datasets from the GEO database to screen DEGs in CC. DEGs from each dataset were then intersected with the m6A data to screen for key genes related to m6A. RBM15 was finally identified to be further studied. The function of RBM15 in CC progression and its specific mechanism were studied.

## Materials and methods

### Microarray data

The GSE9750 and GSE63514 datasets were acquired from the Gene Expression Omnibus (GEO) (https://www.ncbi.nlm.nih.gov/geo/). The GSE9750 dataset (GPL96, [HG-U133A] Affymetrix Human Genome U133A Array) contains 33 primary tumors and 24 normal cervical epithelia, and the GSE63514 dataset (GPL570, [HG-U133_Plus_2] Affymetrix Human Genome U133 Plus 2.0 Array) contains 28 tumor tissue samples and 24 normal tissue samples.

### Screening for DEGs

The GSE9750 and GSE63514 datasets were subjected to analysis by GEO2R (https://www.ncbi.nlm.nih.gov/geo/geo2r) to obtain gene expression data. DEGs were screened according to the p < 0.05 and |logFC| > 0.5. By reviewing the m6A2Target database (http://rm2target.canceromics.org/), we collected m6A-related genes. The Venn diagram tool (http://bioinformatics.psb.ugent.be/webtools/Venn/) was employed to identify the m6A-related DEGs across three datasets: GSE9750, GSE63514, and the m6A2Target datasets.**GO and KEGG enrichment analysis of DEGs**.

Functional enrichment analysis of the DEGs was analyzed using Gene Ontology (GO) and Kyoto Encyclopedia of Genes and Genomes (KEGG) in the DAVID (Database for Annotation, Visualization and Integrated Discovery) database (https://david.ncifcrf.gov/), which is a comprehensive database encompassing genomic, chemical, and systemic functional information [25].

### PPI network construction and m6A-related hub DEGs determination

Using the STRING database (https://string-db.org/) with a confidence score of 0.15, a PPI (protein-protein interaction interaction) network of the DEGs was established. We then screened out the linkage data for proteins associated with m6A and loaded them into the Cytoscape software (version 3. 9. 1) for visualization. Hub modules were identified using the Molecular Complex Detection (MCODE) plug-in. The plug-in of CytoHubba was used to calculate each protein degree of nodes and filtered out hub genes based on connectivity.

### Analysis of m6A-related hub DEGs

Receiver operating characteristic (ROC) curves, Principal component analysis (PCA), and a ridge plot of the hub genes expression abundance were performed by the pROC package, factoextra package, and ggridges package, respectively in R software (version 3.6.0). In the GEPIA2 database (http://gepia2.cancer-pku.cn/), the mRNA expression levels of hub genes were explored in CC tissues. In the Kaplan-Meier Plotter database (https://kmplot.com/analysis/), the effect of RBM15 expression on the survival of CC patients was evaluated. The correlation between RBM15 and CD4 + T cells was analyzed by the TIMER2.0 database (http://timer.cistrome.org/).

### Cell culture

Normal cervical epithelial cell line: HcerEpic, and CC cell line: SiHa and Hela (Icellbioscience, Shanghai, China) were grown in DMEM medium (Gibco, NY, USA) containing 1% penicillin and streptomycin, and 10% fetal bovine serum (Gibco). Cells were placed in an incubator (Thermo Fisher Scientific, Massachusetts, USA) in a humid environment, and the temperature of the incubator was set to 37 °C with a 5% CO_2_ condition.

### Reverse transcription quantitative PCR (RT-qPCR)

Cells were subjected to RNA extraction using TRIzol Reagent (Thermo Fisher Scientific) to obtain the complete RNA. Following the evaluation of RNA samples for quantification and quality using a NanoDrop spectrophotometer (Thermo Fisher Scientific) and standard agarose gel electrophoresis, respectively, the RNA was subjected to reverse transcription into cDNA using a reverse transcription kit (Thermo Fisher Scientific). The PCR quantification was performed using the 7500 real-time PCR System (Thermo Fisher Scientific) with the SYBR Premix Ex Taq reagent (Takara, Dalian, China). The glyceraldehyde-3-phosphate dehydrogenase (GAPDH) was used to normalize the results, and the relative expression levels of the mRNAs were determined using the 2^−ΔΔCT^ method. Supplementary Table 1 contains the primer sequences used in the study.

### Cell transfection

The siRNA transfection was utilized to transiently knock down RBM15 expression. Three types of the small interfering RNA (siRNA) aimed at human RBM15 and the non-targeting control siRNA, were procured from Shanghai Integrated Biotech Solutions Co., Ltd. (Shanghai, China). A total of 1 × 10^5^ cells/well SiHa and Hela cells were incubated in a 24-well plate until they attained a confluence of 70–90%. Subsequently, they were subjected to transfection with 50 nmol siRBM15 and negative control (NC), with the Lipofectamine 3000 reagent (Thermo Fisher Scientific), according to the manufacturer’s instructions. The effectiveness of silencing RBM15 was validated by RT-qPCR analysis. Supplementary Table 1 displays the sequences of the siRNAs used.

### Cell counting kit-8 (CCK8) assays

The transfected 100 µL cell suspension (The density was 1 × 10^5^ cells/well) was seeded into 96 well plates. Subsequently, 10 µL of CCK8 solution (Solarbio, Beijing, China) was added to each well at 0, 24, 48, 72, and 96 h, respectively. Following a 2-hour incubation, the absorbance at 450 nm was measured with a microplate reader (DALB, Shanghai, China).

### Scratch wound healing

The density of cells in each group was adjusted to 5 × 10^5^/well and then inoculated into six-well plates. When the cells had reached 80% confluence, a 10 µL pipette was used to draw a horizontal line on the cell surface. Images were captured immediately after scratching 24 h later. The migration distances and migration rates were calculated using the Image J program.

### Transwell assays

Following transfection for 48 h, we used serum-free medium to resuspend the cells. After coating the transwell chambers with matrigel gel (Becton, Dickinson and Company, New York, USA), in the upper chamber, cells were added, and in the lower chamber, 600 µL of medium supplemented with 15% fetal bovine serum was added. Following a 24-hour incubation, cells were fixed in 4% paraformaldehyde at room temperature for 15 min before being stained with 0.1% crystal violet at 37 °C for 30 min. Finally, cells were observed and photographed using a light microscopy (OLYMPUS, Tokyo, Japan).

### Mice tumor xenograft model experiments

Six-week-old, female BALB/c nude mice (SPF (Beijing) Biotechnology Co, Ltd, Beijing, China) were randomly allocated into two groups (n = 6 in each group). To establish a tumor xenograft model, 0.1 ml Hela cell (transfected with RBM15 shRNA or control shRNA) suspension at a concentration of 1 × 10^7^/ml was injected subcutaneously into the right axillary region of mice. The growth of the xenograft tumor was checked weekly after the first administration, and the tumour diameter was determined by a vernier caliper. The calculation of the tumor volume was performed by the formula V = 1/2ab^2^ (a is the maximum tumour diameter, and b is the minimum tumour diameter) and continuously measured for 4 weeks. Mice were executed after the last measurement, and the tumors were weighed. Our Animal Ethics Committee approved all the above experiments.

### Western blot analysis

Proteins extracted from tumor tissues and cells were lysed using RIPA lysis buffer containing a protein inhibitor (Solarbio). The protein concentrations of the samples were determined by the BCA assay (Thermo Fisher Scientific). The protein was loaded onto a sodium dodecyl sulfate–polyacrylamide gel to complete separation. Then they were transferred to the polyvinylidene difluoride (PVDF) membranes (Roche, Basel, Switzerland), which was conducted at a current of 200 mA for approximately 50–60 min, depending on the size of the proteins. The PVDF membrane was subjected to blocking in 5% bovine serum albumin (BSA; Thermo Fisher Scientific) at room temperature for 1 h and then was incubated overnight at 4 °C with primary antibody. Following three washes in TBST (10 min each), the membrane was incubated with the secondary antibody (1:5000, Abcam) at room temperature for 2 h. Subsequently, the membrane was washed three times with TBST. The immunoblots were visualized using an ECL luminescent solution (Amersham, Little Chalfont, UK) as per the instructions provided by the manufacturer. The resulting immunoblots were then analyzed using Image J software. And the antibodies utilized were as listed below: anti-RBM15 (1:2000, Abcam),, P-JAK2 (1:2000, Abcam), JAK2 (1:2000, Abcam), P-STAT3 (1:2000, Abcam), STAT3 (1:2000, Abcam), P-STAT5 (1:2000, Abcam), STAT5 (1:2000, Abcam), and anti-β-actin (1:2000, Abcam).

### Mechanisms of RBM15 affecting CC progression

We performed a screening of the top 1000 genes related to CC using the GeneCards database (https://www.genecards.org), taking into consideration their relevance scores. Furthermore, the Coxpresdb database (https://coxpresdb.jp/) was utilized to select the top 1000 genes that were co-expressed with the RBM15 gene. By creating a Venn diagram, we identified overlapping genes between the two databases. Then KEGG enrichment analysis of overlapping genes was performed by DAVID database.

### Statistical data analysis

The data obtained from the experiment were processed using Graphpad Prism 8.0 statistical software, and the plots were generated using R software (version 3.6.1). The measurement data were expressed as mean ± standard deviation (SD). For the comparison of two groups, the T-test was used, while analysis of variance (ANOVA) was employed to compare multiple groups. Following ANOVA analysis, Tukey’s multiple comparisons test was used to compare two groups. A p-value less than 0.05 was considered statistically significant.

## Results

### Identification of DEGs in CC

**Fig. 1:**
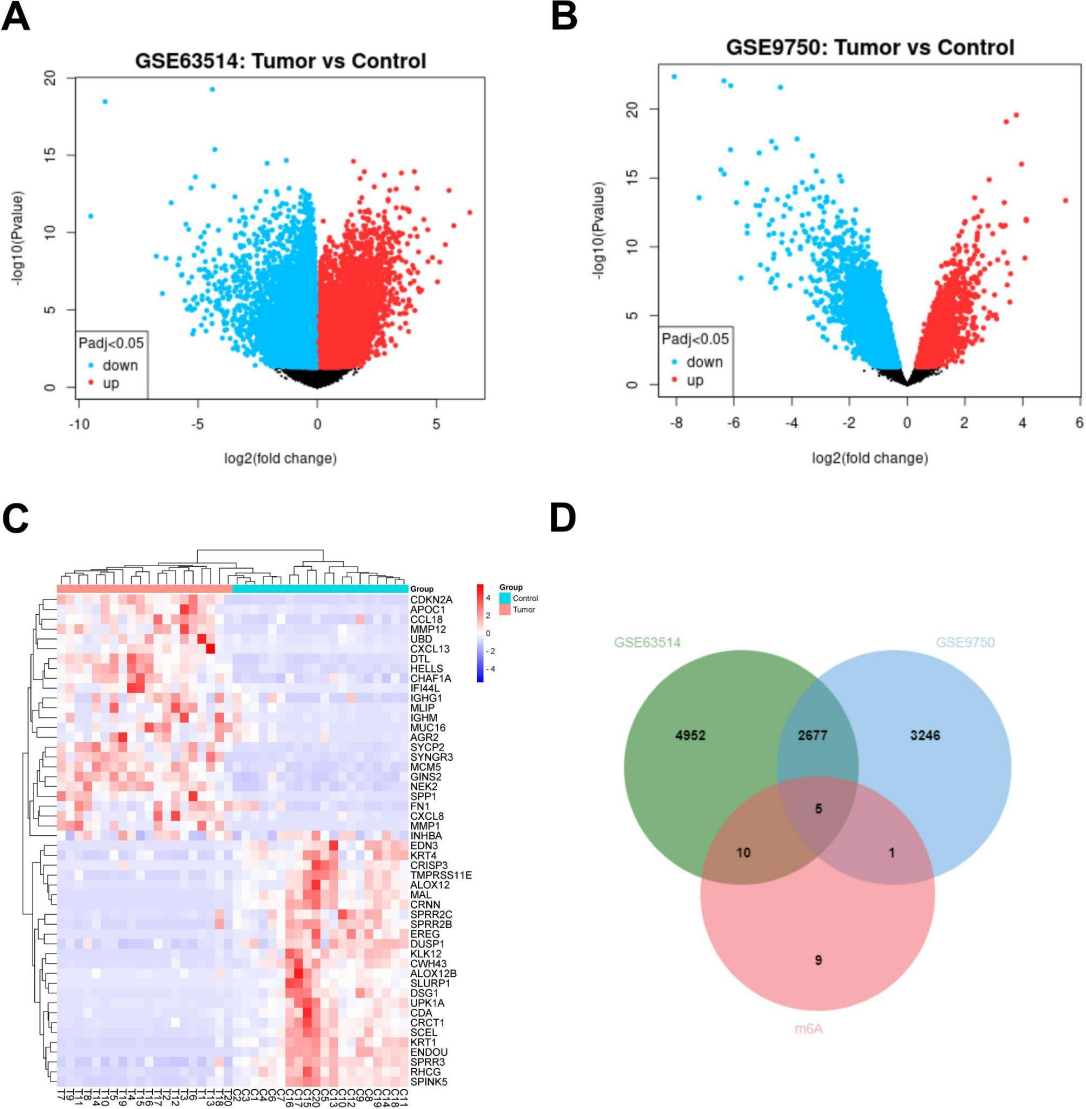
Identification the DEGs in CC based on GEO database. (**A**) The volcano plots display the distribution of DEGs in the GSE63514 dataset. (**B**) The volcano plots of GSE9750 dataset. (**C**) The Heatmap displays the top 25 upregulated and top 25 downregulated DEGs that overlap between GSE9750 and GSE63514. (**D**) The Venn diagram of the overlapping m6A-related DEGs.

In the GEO database, the GSE9750 and GSE63514 datasets were selected to obtain DEGs by the GEO2R tool, and mapped the volcano (Fig. 1A and B). The read counts for each sample were normalized. The results revealed that the median values for each sample were near to identical, indicating that the GSE7670 and GSE63514 datasets fitted the criteria for further study (Supplementary Fig. 1). A total of 7644 DEGs were identified in the GSE63514 dataset and 5929 DEGs in the GSE9750 dataset (Supplementary Tables 2 and 3). The 25 overlapping most highly up- and down-regulated DEGs from the GSE9750 and GSE63514 datasets (Supplementary Table 4) were filtered out and plotted as a Heatmap using R software (Fig. 1C). According to the Venn diagram, there were 2682 DEGs that were commonly identified in both the GSE9750 and GSE63514 datasets. By reviewing the m6A2Target database, we collected 24 m6A-related genes (Supplementary Table 5) to the intersection with the overlapping DEGs for screening m6A-related DEGs, and five m6A-related DEGs were selected (Fig. 1D; Supplementary Table 6).

### GO and KEGG analysis of the overlapping DEGs

**Fig. 2:**
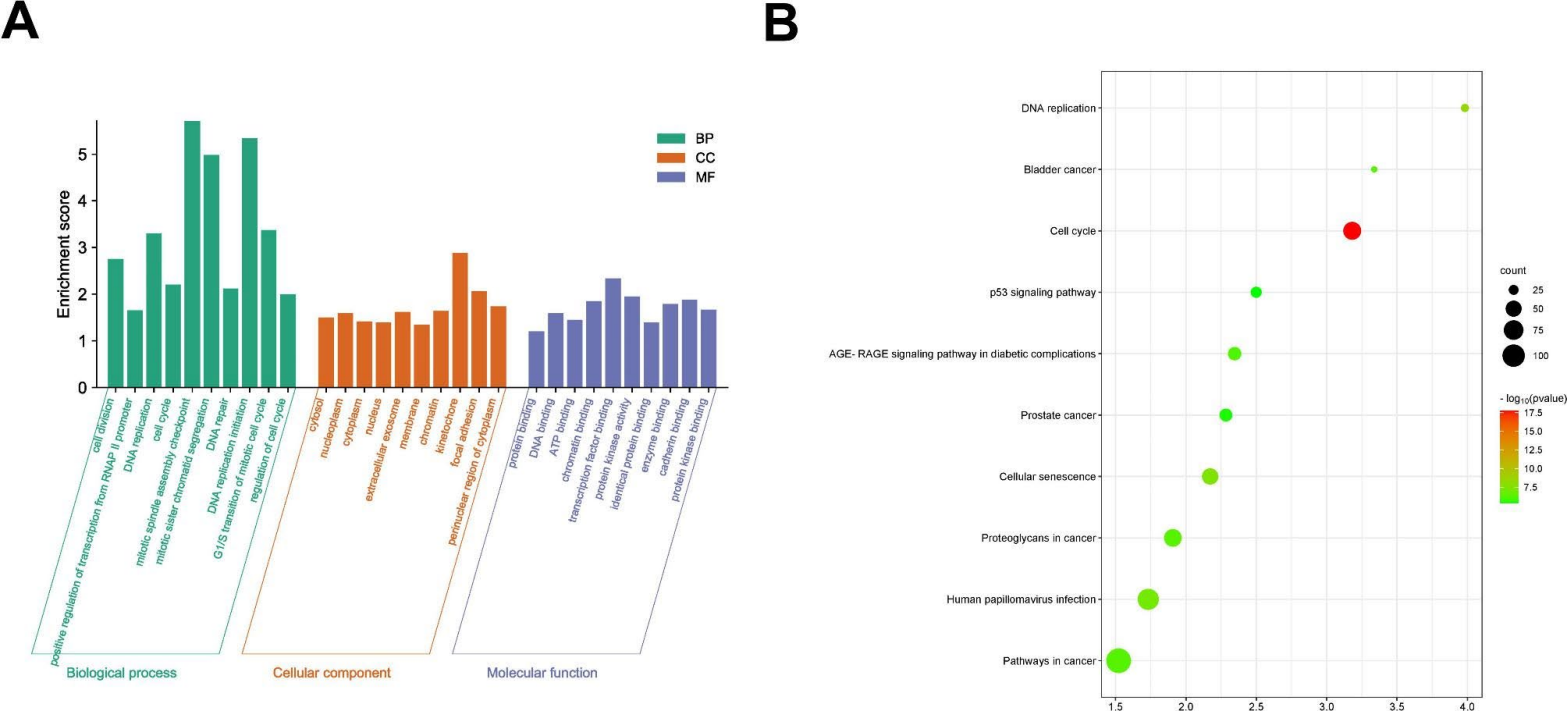
GO terms and KEGG pathway were analyzed for the overlapping DEGs. (**A**) A bar chart to visualize the top 10 GO terms. (**B**) A bubble plot of the KEGG pathway ranked in the top 10

We uploaded the overlapping DEGs to the DAVID database for GO and KEGG pathway analyses. GO functional enrichment analysis is divided into 3 categories, including MF (molecular function), BP (biological process), and CC (cellular component). The bar chart demonstrated the enrichment analysis results that ranked in the top 10 for every individual GO term, according to the p-value. In the MF group, the DEGs were predominantly involved in pathways related to cancer, human papillomavirus infection, and proteoglycans in cancer. In the BP, the DEGs were mainly involved in the membrane, nucleus, and cytoplasm. Regarding the CC category, the DEGs were found to be predominantly enriched in processes such as positive regulation of transcription from RNA polymerase II promoter, regulation of cell cycle, and DNA repair (Fig. 2A). Using the p-values as a basis, we filtered for the top 10 KEGG pathways and visualized them in a bubble plot (Fig. 2B). The findings demonstrated that the DEGs were primarily enriched in functions related to protein binding, identical protein binding, and ATP binding.

### Construction of a protein-protein interaction (PPI) network and analysis of modules comprising m6A-related genes

**Fig. 3:**
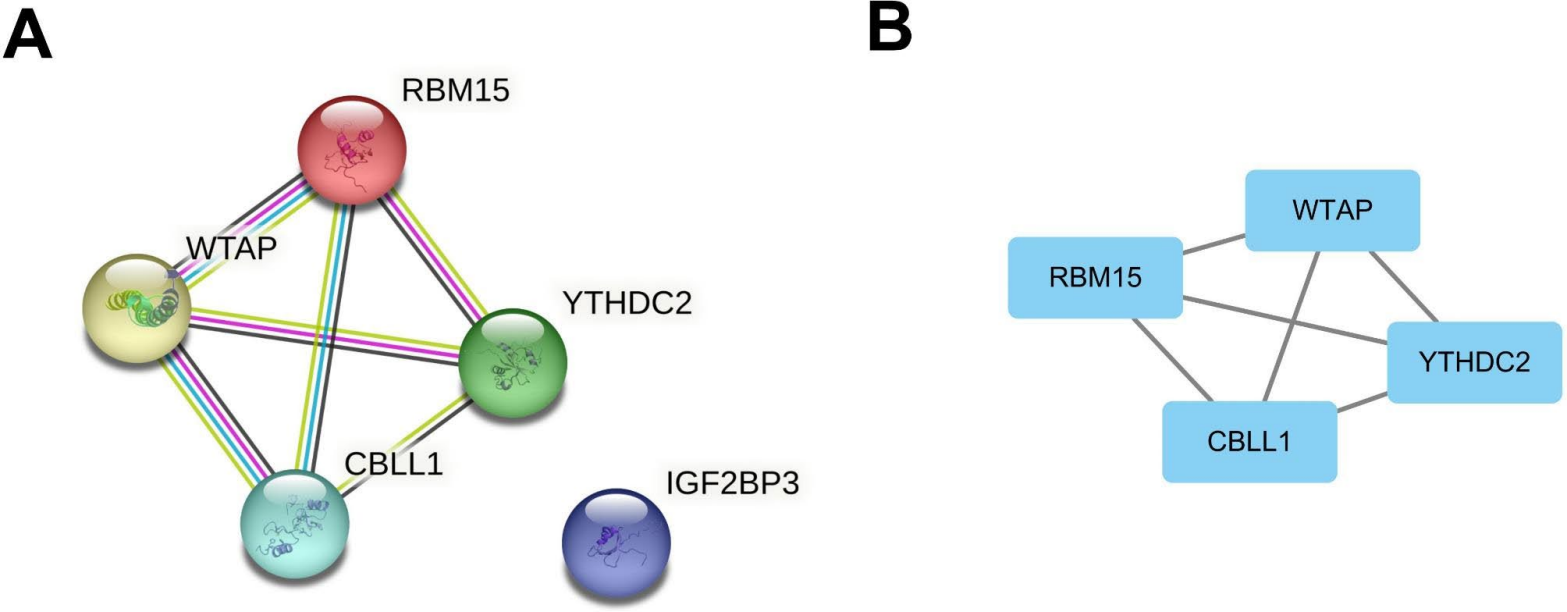
Screening of m6A-related key modules in the GSE9750 and GSE63514 datasets. (A). Panoramic view of the PPI network consisting of m6A-related DEGs. (B). Hub genes identified in m6A-related DEGs based on MCODE analysis

A PPI network was created using the m6A-related DEGs (Fig. 3A). Then a highly interconnected cluster from the PPI network was determined using the MCODE in the Cytoscape software and four hub genes were obtained: WTAP, RBM15, CBLL1, and YTHDC2 (Fig. 3B).

### Bioinformatics analysis was performed to identify hub genes

**Fig. 4:**
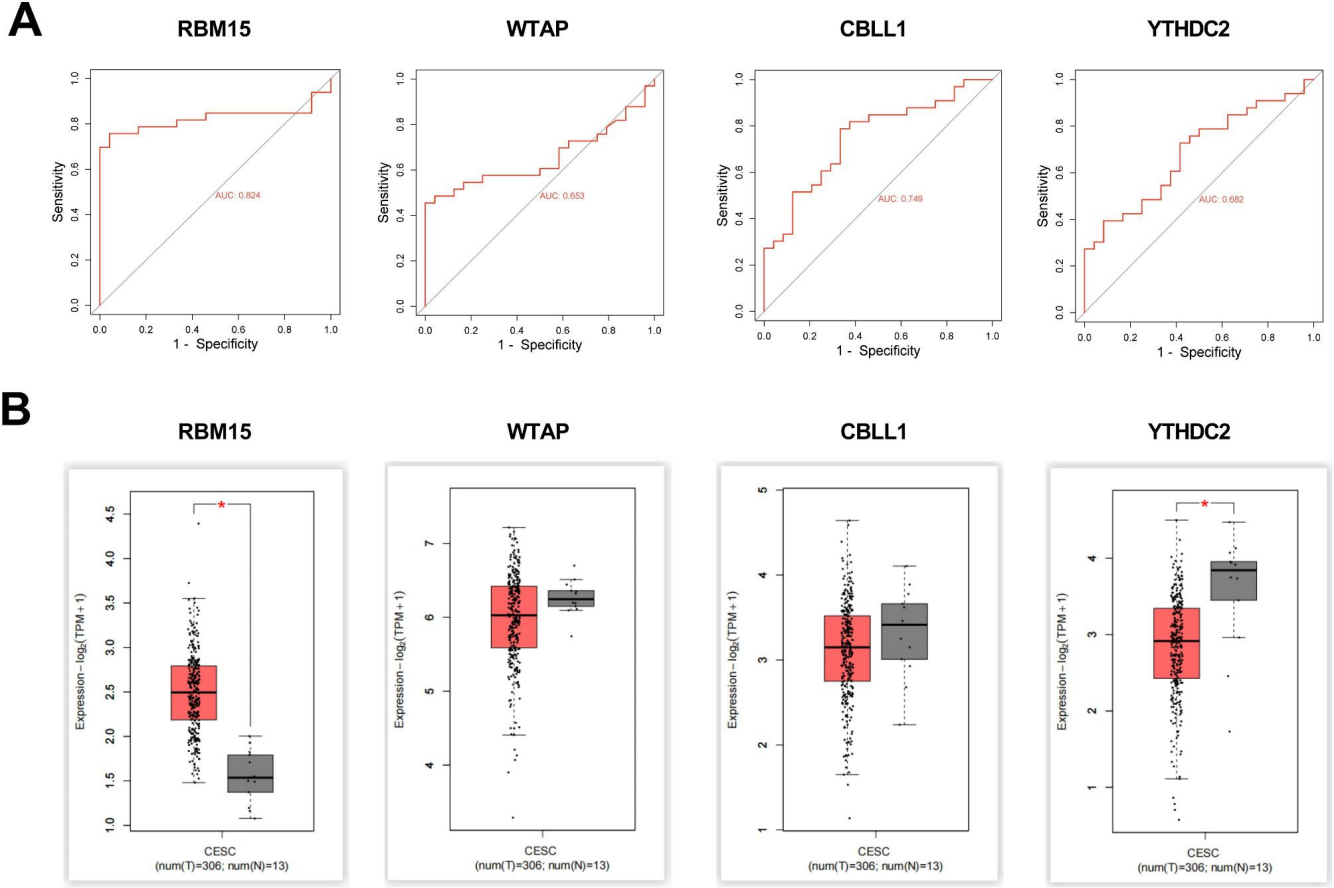
ROC curve and gene expression analysis of hub genes. (**A**) ROC curve analysis, from left to right, for RBM15, WTAP, CBLL1 and YTHDC2. (**B**) Differential expression of hub genes in tumor and non-tumor tissues in CC was analyzed in the GEPIA2 database, and from left to right, for RBM15, WTAP, CBLL1 and YTHDC2. *P < 0.05 vs. normal tissues

ROC curves were utilized to assess the precision of the four hub genes as CC biomarkers (Fig. 4A). The results showed that RBM15 had the highest AUC value (0.824), meanwhile, the AUC values for WTAP, CBLL1, and YTHDC2 were 0.653, 0.749, and 0.682, respectively. The ridge plot of four hub genes expression showed that RBM15 and WTAP had high expression levels (Supplementary Fig. 2). In addition, hub genes expressions in CC were explored in the GEPIA2 database. The findings indicated a significant increase in RBM15 expression in tumor tissues compared to non-tumor tissues. Conversely, the results demonstrated a significant decrease in YTHDC2 expression in tumor tissues compared to normal tissues. (Fig. 4B). Based on the above analysis, we finally determined RBM15 as the object of follow-up research.

### Survival and immune infiltration analysis of RBM15

The results indicated a negative correlation between RBM15 expression and the survival of CC patients. The higher the expression of RBM15, the shorter the survival period of patients (Supplement Fig. 3A). In the TIMER2.0 database, there was a positive association between RBM15 and CD4 + T-cell infiltration in uterine corpus endometrial carcinoma (CESC), but the correlation coefficient was only 0.156 (Supplement Fig. 3B). Besides, there was a positive correlation between RBM15 expression and CD4 + T cell infiltration in a variety of tumors by different algorithms such as TIMER, EPIC, MCPCOUNTER, CIBERSORTT and XCELL (Supplement Fig. 3C).

### Hub gene expression validation

**Fig. 5:**
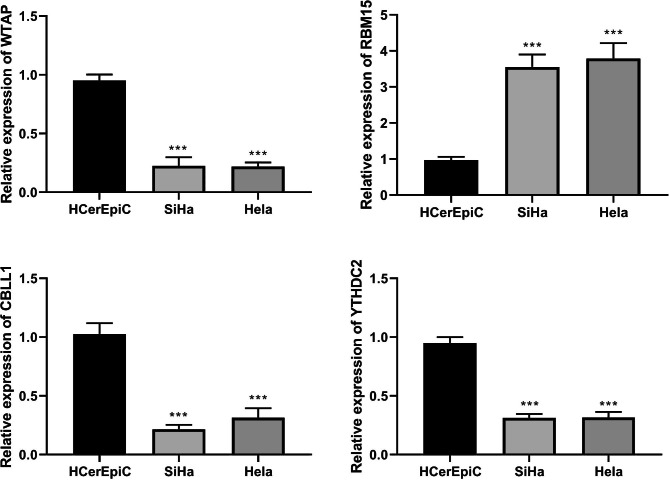
Detection of key m6A-related genes expression in HCerEpic cells and SiHa and Hela cells by RT-qPCR. ***P < 0.001 vs. HcerEpic cells

We then examined the WTAP, RBM15, CBLL1, and YTHDC2 expressions in CC cells by RT-qPCR. According to the results, RBM15 expression was substantially increased in SiHa and Hela cells compared with HCerEpic cells. In contrast, WTAP, CBLL1, and YTHDC2 expressions were significantly downregulated (Fig. 5).

### Knockdown of RBM15 inhibits proliferation, invasion and migration of CC cells

**Fig. 6:**
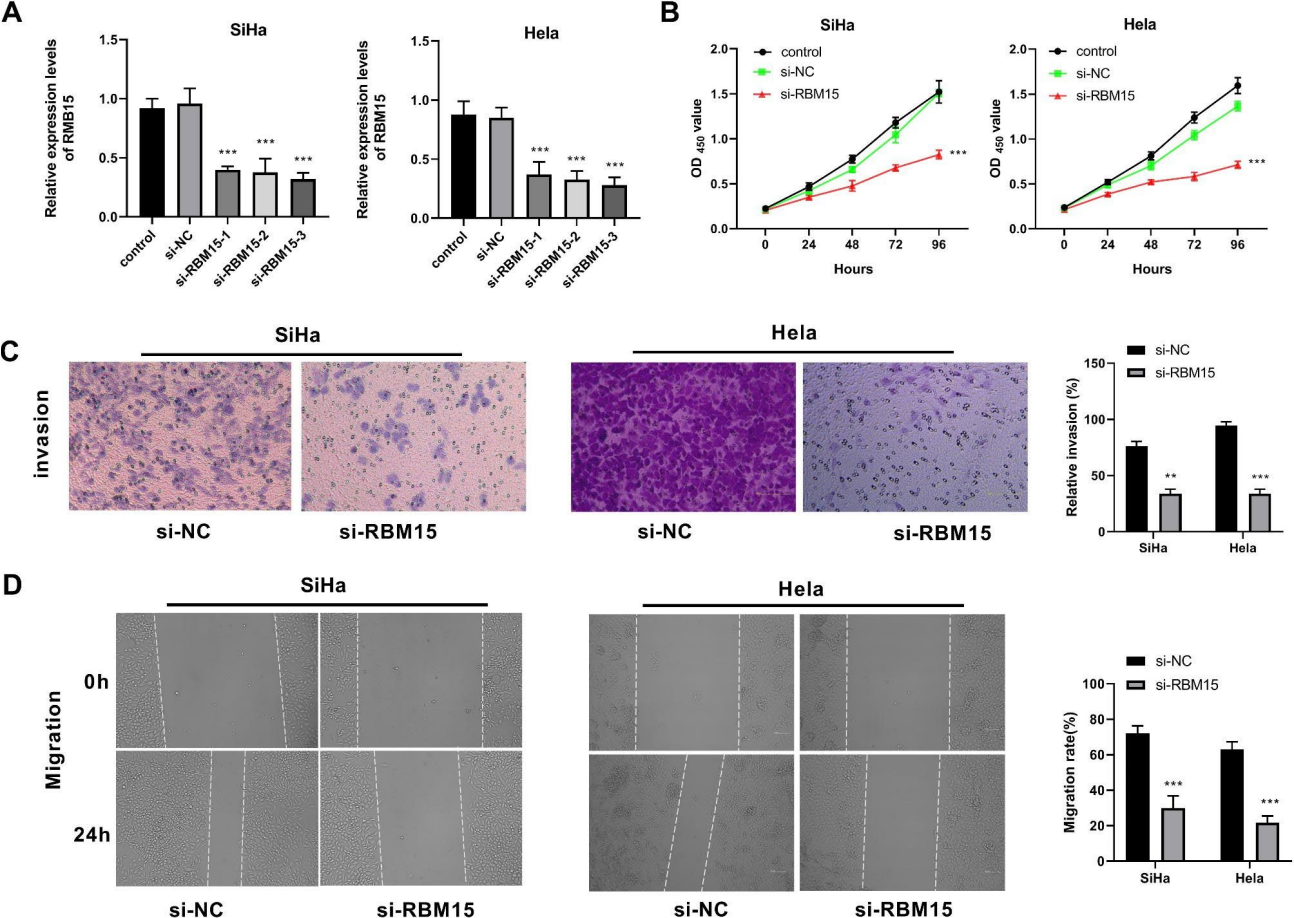
Knockdown of RBM15 inhibits proliferation, invasion, and migration of CC cells. (**A**) Transfection efficiency was detected using the qRT-PCR in SiHa and Hela cells. (**B**) CCK-8 detected the function of RBM15 on SiHa and Hela cell proliferation. (**C-D**) Transwell and wound healing assays was used to detect the effect of RBM15 on the invasion and migration of SiHa and Hela cells. **P < 0.01 vs. si-NC; ***P < 0.001 vs. si-NC.

To further verify the role of RBM15 on CC cells, we knocked down RBM15 using siRNA. Figure 6 A showed the RBM15 expression in the si-RBM15-1, si-RBM15-2, and si-RBM15-3 groups. The si-RBM15-3 groups had the highest knockdown efficiency, so we applied si-RBM15-3 for our subsequent experiments. The results of the CCK-8 assay showed that the proliferation ability of SiHa and Hela cells was significantly reduced in the si-RBM15 group (Fig. 6B). The results from the Transwell and wound healing assays indicated that SiHa and Hela cells in the si-RBM15 group showed significantly reduced invasion and migration abilities compared to those in the si-NC group (Fig. 6C-D).

### Knockdown of RBM15 inhibits the growth of CC in vivo

**Fig. 7:**
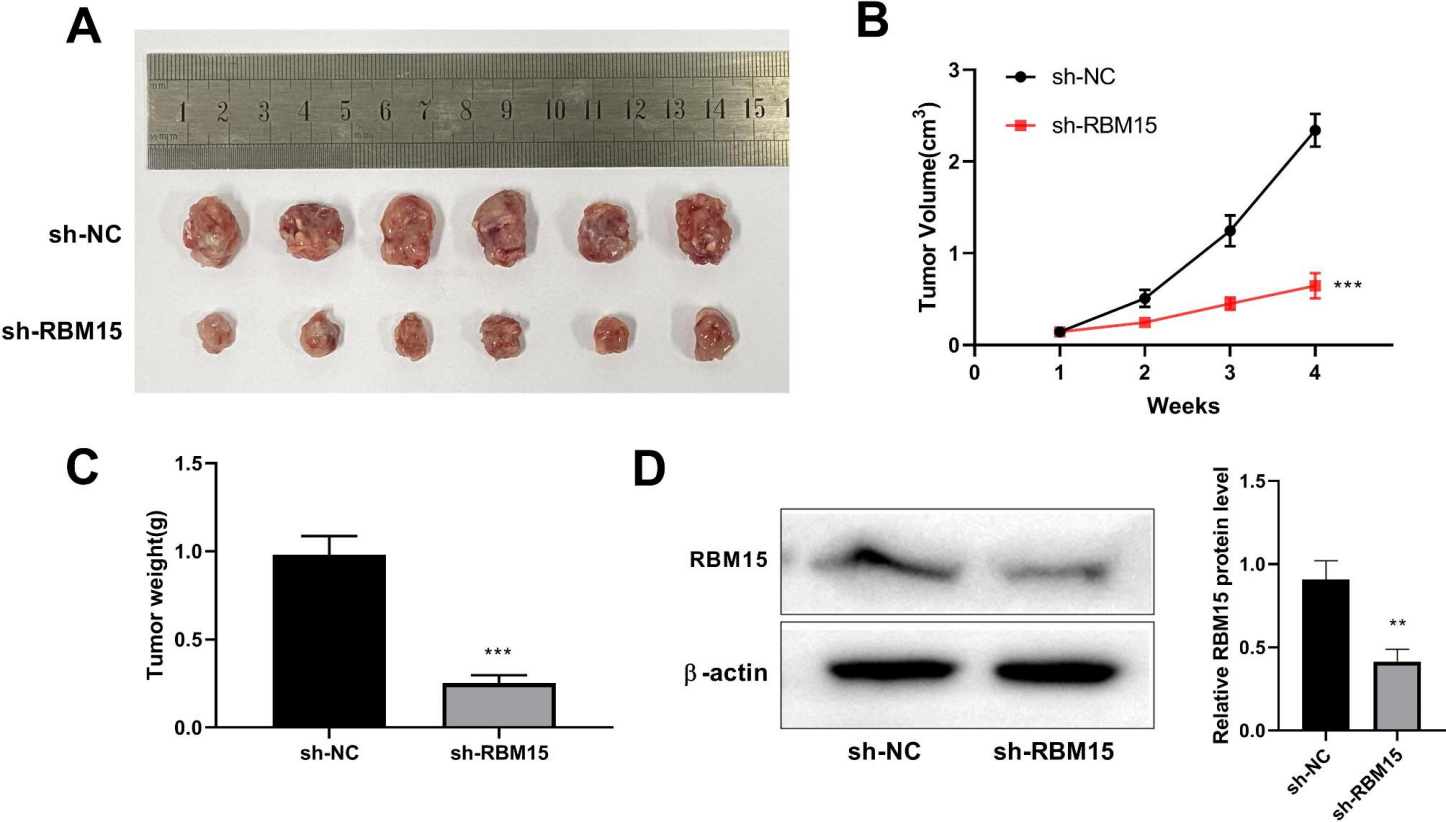
Exploring the function of RBM15 on CC growth. (**A**) Image depicting the nude mice nude mice that were injected with Hela cells. (**B**) The tumor volume growth curve was measured in RBM15 knockdown cell and its control group. (**C**) The tumor weight growth curve was measured in the RBM15 group cell and its control group. (**D**) Western blotting determined the protein silencing efficiency of RBM15. **P < 0.01 vs. sh-NC; ***P < 0.001 vs. sh-NC.

Then we verified the of RBM15 in CC growth by establishing mouse xenograft tumor models. The results indicated that the sh-RBM15 group exhibited significantly lower volume and weight of transplanted tumors compared to the sh-NC group (Fig. 7A-C). Figure 7D showed a significant reduction in RBM15 protein expression.

### Silencing RBM15 inhibits the expression levels of proteins related to the JAK-STAT signaling pathway

**Fig. 8:**
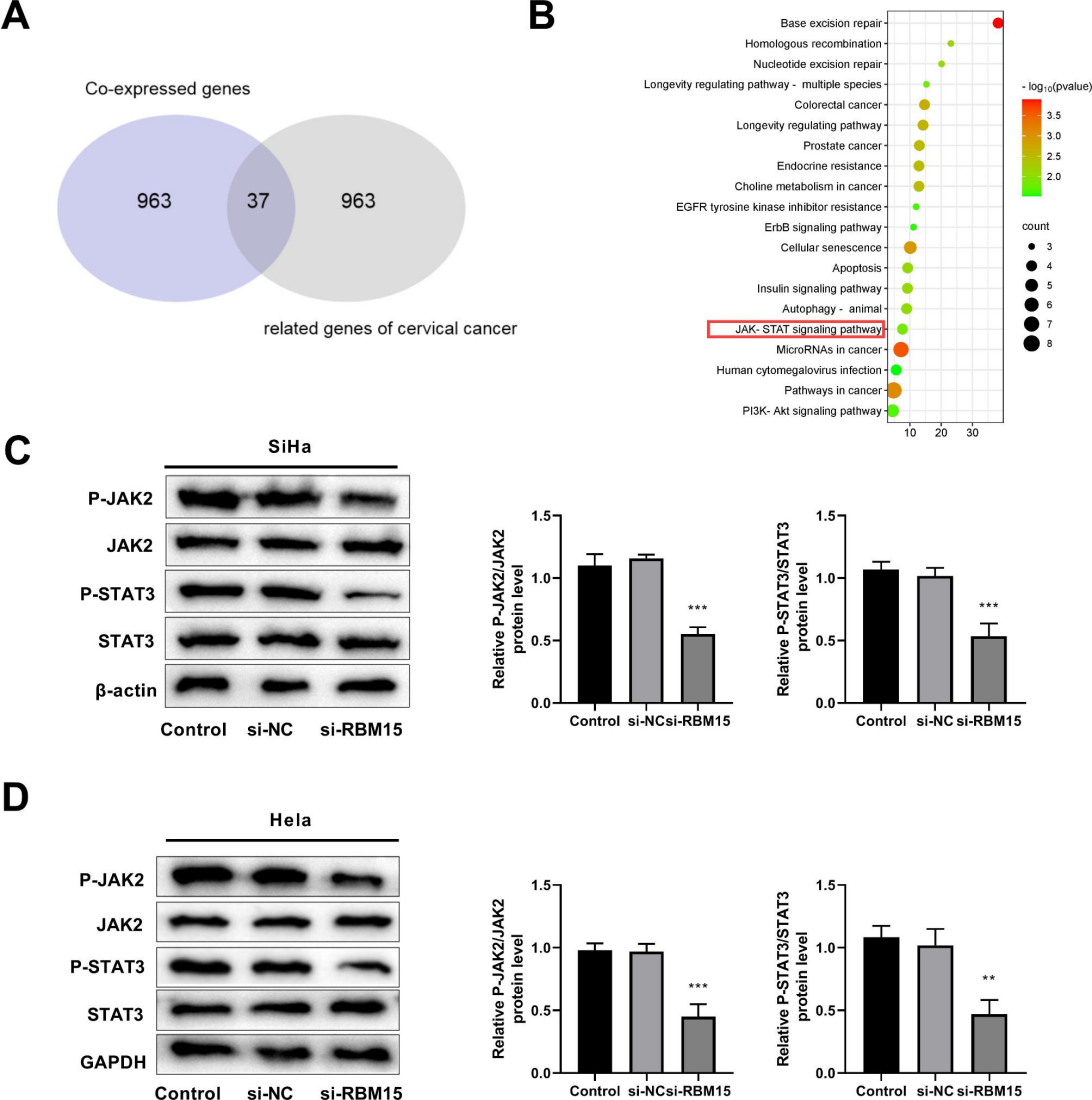
Silencing RBM15 inhibits the phosphorylation levels of proteins related to the JAK-STAT signaling pathway. (**A**) Venn diagram of RBM15 co-expressed genes (top 1000) and CC-associated genes (top 1000). (**B**) KEGG enrichment analysis of overlapping genes. (**C**) Western blot was used to detect the expression levels of proteins with relevance to the JAK-STAT pathway in SiHa cells. (**D**) Western blot was used to detect the expression levels of proteins with relevance to the JAK-STAT pathway in Hela cells. ***P < 0.001 vs. control

Then, the molecular mechanisms of RMB15 affecting CC progression was explored. We screened CC-related top 1000 genes using the GeneCards database based on their relevance score. Additionally, the Coxpresdb database was employed to select the top 1000 genes co-expressed with the RBM15 gene. By drawing a Venn diagram, we identified 37 overlapping genes between the two databases (Fig. 8A). Then a KEGG enrichment analysis performed on the 37 overlapping genes, and the results revealed that they were significantly enriched in the JAK-STAT signaling pathway (Fig. 8B). Next, Western blot was used to detect the expression levels of JAK-STAT-signaling pathway-related proteins in SiHa and Hela cells transfected with siRBM15. The results showed that knockdown of RBM15 significantly decreased the proteins expression of P-JAK2/JAK2, P-STAT3/STAT3, and P-STAT5/STAT5 in SiHa and Hela cells (Fig. 8C and D).

## Discussion

Approximately 84% of all CC cases occur in countries with poor human development indexes. The majority of patients are diagnosed with CC at an advanced stage, and the prognosis is extremely poor, even in developed countries [26]. Therefore, it is essential to find biomarkers and therapeutic targets for CC. In the present study, RBM15 was identified as an independent prognostic factor for CC, and the knockdown of RBM15 significantly inhibited the proliferation, invasion, and migration of CC cells and the JAK-STAT signaling pathway.M6A modification appears to be dynamic and reversible as it is regulated by methyltransferases, and demethylases. M6A recognition proteins play a crucial role in the function of m6A modifications [27]. The m6A-associated proteins regulate multiple tumor progression. Upregulation of KIAA1429 expression in hepatocellular carcinoma induces m6A modification on pre-mRNA of GATA3, leading to increased tumor cell proliferation and metastasis [28]. YTHDF1 is overexpressed in ovarian cancer and knockdown of YTHDF1 inhibits tumor progression [29]. Several studies have elucidated the important role of m6A-associated proteins in CC development. METTL3 is overexpressed in CC and silencing of METTL3 can reduce cancer cell viability, promote apoptosis and increase cisplatin sensitivity [30]. METTL14 expression is upregulated in CC, and knocking down METTL14 significantly inhibited tumor growth and invasion [31]. YTHDF1 regulates RANBP2 translation efficiency in an m6A-dependent manner, promoting CC cell growth, migration and invasion [32]. In this study, our results showed that RBM15 was noticeably upregulated in SiHa and Hela cells, while WTAP, CBLL1, and YTHDC2 expressions were significantly downregulated. Research has shown that WTAP is located in the nuclei and has a significant correlation with overall survival in CC patients [33]. The prognosis of UCEC is influenced by missense mutations in YTHDC2 [34]. The high expression of RBM15 in CC suggested a potential involvement as an oncogene, which had sparked significant interest among us. So we subsequently focused on the role of RBM15 in CC.

RBM15 belongs to the SPEN (split-end) protein family, which can be found on chromosome 1p13.3 [35]. It has been reported that RBM15 directly binds to U-rich regions on mRNA to attract the methyltransferase complex to its target transcripts [36]. Based on the current study, RBM15 was found to be an oncogenic factor in most tumors. RBM15-MKL1 fusion resulting from chromosomal translocation t(1;22)(p13;q13) can lead to acute megakaryocytic leukemia [37]. RBM15 enhances the growth, metastasis, and infiltration of clear cell renal cell carcinoma [38]. In addition, RBM15 interacts with circ-CTNNB1 to enhance the expression of HK2, GPI and PGK1 in an m6A modification-dependent manner, leading to the promotion of the glycolytic process and the development of osteosarcoma [39]. In our study, we discovered that RBM15 functions as a tumor oncogene in CC, and knockdown of RBM15 inhibits cell proliferation, migration, and invasion, as well as tumor growth in vivo.

Subsequently, we conducted further investigations to understand the molecular mechanisms underlying the impact of RBM15 on the progression of CC. Our findings revealed a significant correlation between RBM15 and the JAK/STAT signaling pathway in CC. A previous bioinformatics study has shown that immune-related genes of CC are actively involved in the JAK-STAT pathway, which is consistent with our study [40], suggesting that the JAK-STAT signaling pathway plays an important role in CC progression. Moreover, the study has shown that HPV exploits the JAK/STAT signaling pathway to circumvent the immune system and stimulate cellular proliferation, thereby establishing viral persistence and propelling the progression of cancer [41]. The progression of CC is impeded through the regulation of the JAK/STAT3 signaling pathway by silencing ITGB6 [42]. And the involvement of STAT3 and its associated key genes, highlighting a significant pathogenic role of the JAK/STAT3 pathway in HPV-mediated CC [43]. In our study, we found that knockdown of RBM15 significantly inhibited the protein expression of P-JAK2, JAK2, P-STAT3, STAT3, P-STAT5, and STAT5 in CC through further functional analysis. The evolutionary conservation of the JAK/STAT signaling pathway comprises essential constituents, namely ligand-receptor complexes, JAKs, and STATs [44]. Upon receptor-ligand coupling, which leads to receptor dimerization/oligomerization, the associated JAK proteins phosphorylate themselves and trans-phosphorylate the tyrosine residues on the receptors, providing docking sites for the SH2 domains of the latent cytoplasmic STAT molecules [45]. JAK2 is a non-receptor tyrosine kinase, which can be phosphorylated by members of both the gp130 receptor family and the class II cytokine-receptor family [46]. Synergistic suppression of the survival and proliferation of CC cells is regulated through the combined inhibition of JAK1/2 and DNMT1 using newly identified small-molecule compounds [47]. STAT3 and STAT5 are both members of the STAT family [48]. STAT3 and STAT5 activity is frequently elevated aggressive subtypes of cancer, making them valuable prognostic indicators [49–51]. The pivotal role of STAT3 in tumor development stems from its capacity to regulate the transcription of genes involved in various key processes, such as the cell cycle (CCND1, CCNE1), metabolism (OCT1, HIF1A), and cell survival (BCL2, BCLXL, and HSP70) [52]. Interference with STAT3 using siRNA leads to an enhancement in the sensitivity of CC cells to cisplatin [53]. And in 90% of the CC patients, HPV infection is detected and shows a significant association with overexpression of STAT5 [54]. Therefore, combined with the above findings, we hypothesized that RMB15 was likely to influence the development of CC by regulating the JAK-STAT signaling pathway, and that the RBM15/JAK2/STAT3/STAT5 axis hold promise as a potential therapeutic target for CC treatment.

## Conclusion

Finally, the present study aimed to identify m6A-related DEGs that may contribute to the development of CC. And RBM15 has the potential to be developed as a diagnostic biomarker for CC. Knockdown of RBM15 in CC has been shown to suppress tumor cell proliferation, invasion, and migration as well as the JAK-STAT signaling pathway. Our study revealed an important correlation between the the JAK-STAT signaling pathway and m6A in CC.

## Electronic supplementary material

Below is the link to the electronic supplementary material.

Supplementary Material 1

Supplementary Material 2

Supplementary Material 3

Supplementary Material 4

Supplementary Material 5

Supplementary Material 6

Supplementary Material 7

Supplementary Material 8

## Data Availability

All data in the manuscript is available through the responsible corresponding author.
