# Supplementary material for: Knockdown of RBM15 inhibits tumor progression and the JAK-STAT signaling pathway in cervical cancer
Source: BMC Cancer. 2023 Jul 20;23:684. doi: 10.1186/s12885-023-11163-z (PMC10360283; doi:10.1186/s12885-023-11163-z)

Supplementary Fig 1 The read counts for each sample were normalized in GSE63514 and GSE9750.


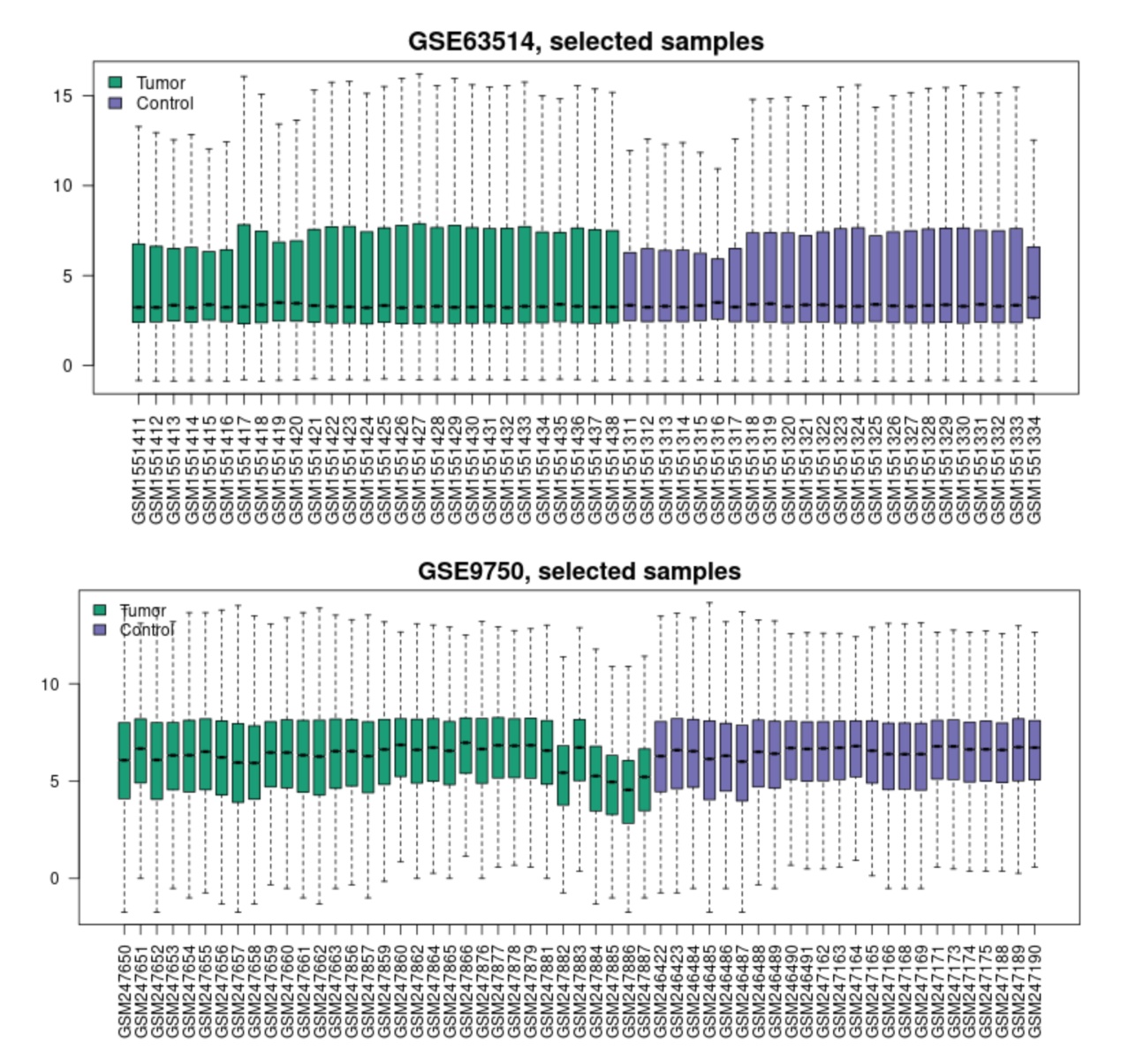


Supplementary Fig 2 PCA and expression ridgeline analysis of hub genes. A. PCA analysis of hub gene, in which the axes PC1 and PC2 are the first and second principal components (explanation rate of differences by potential variables). Dots represent samples, and different colors indicate different groupings. B. Hub gene ridge line plot. Horizontal coordinates represent gene expression, and vertical coordinates represent sample abundance of the corresponding expression.


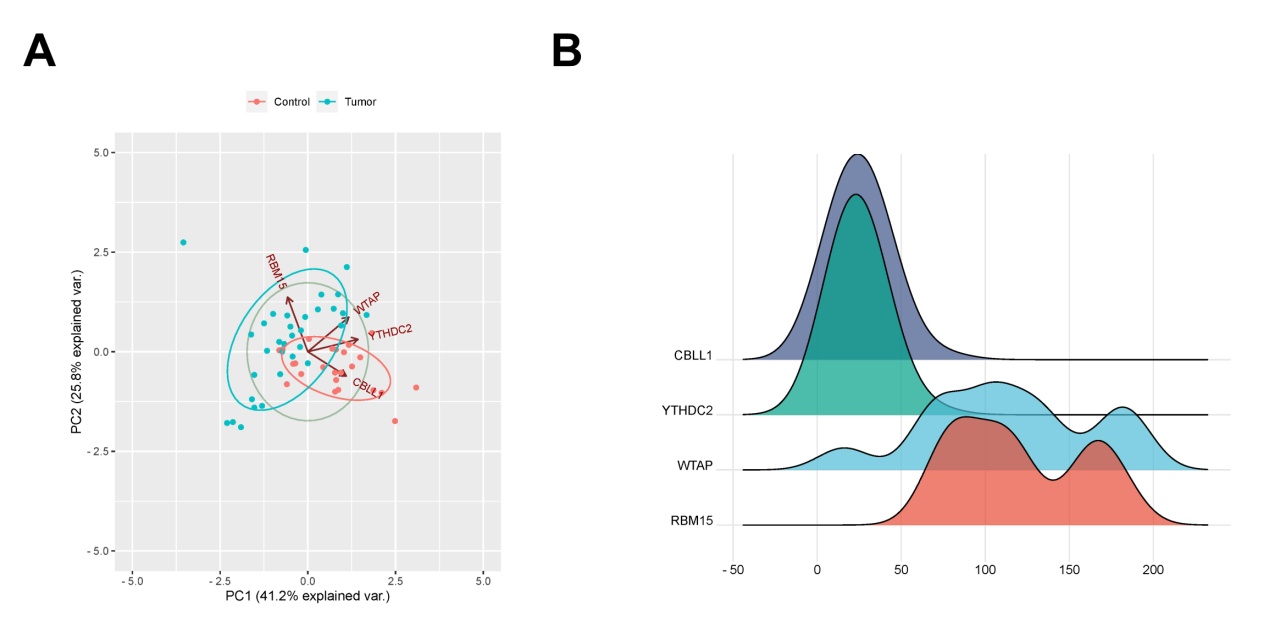


Supplementary Fig 3 Investigation of the association between RBM15 expression and immune infiltration and survival in CC. A. The Kaplan-Meier Plotter database was used to analyze the correlation between RBM15 expression and survival of CC patients. B-C. Correlation between RBM15 expression levels and CD4 + T cell immune infiltration.
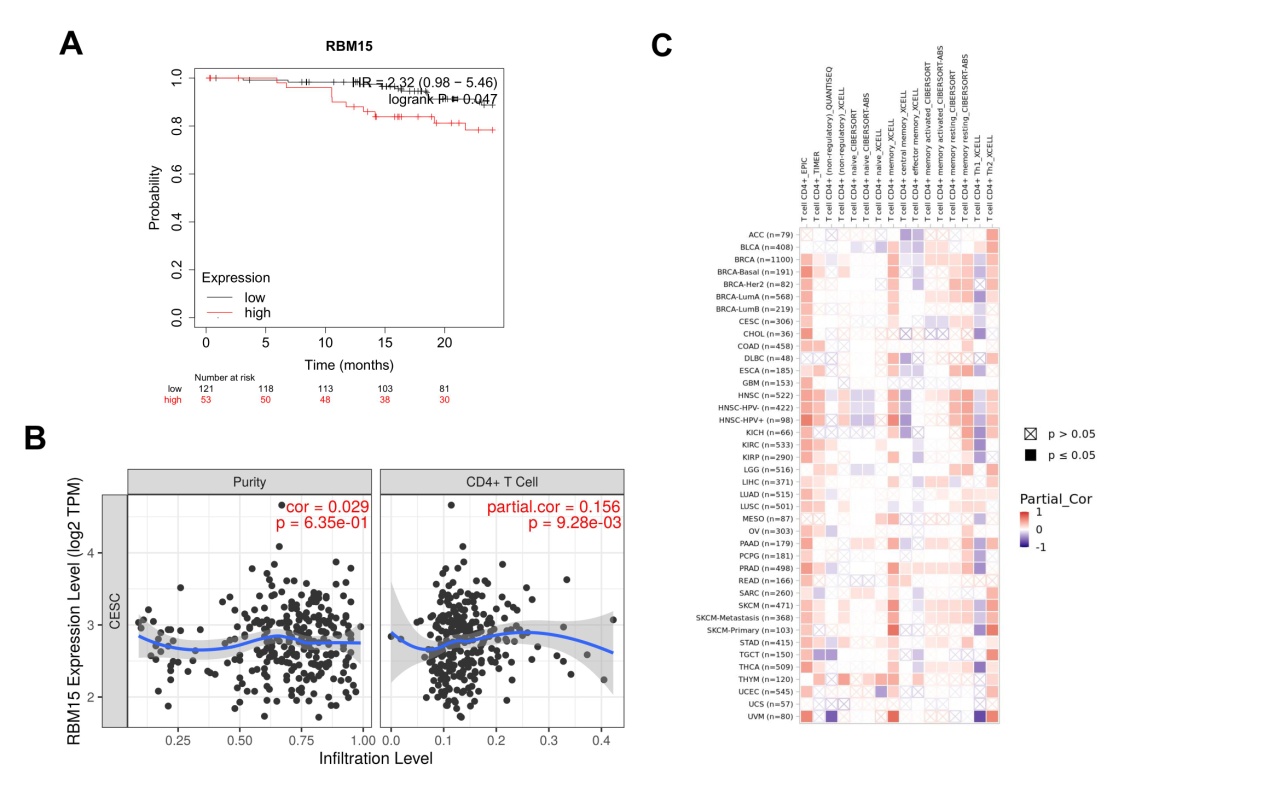

Supplement: Supplementary file 1 — Supplementary Material 1 [file 12885_2023_11163_MOESM1_ESM.docx]
