# Supplementary material for: Knockdown of RBM15 inhibits tumor progression and the JAK-STAT signaling pathway in cervical cancer
Source: BMC Cancer. 2023 Jul 20;23:684. doi: 10.1186/s12885-023-11163-z (PMC10360283; doi:10.1186/s12885-023-11163-z)

Figure 7D RBM15

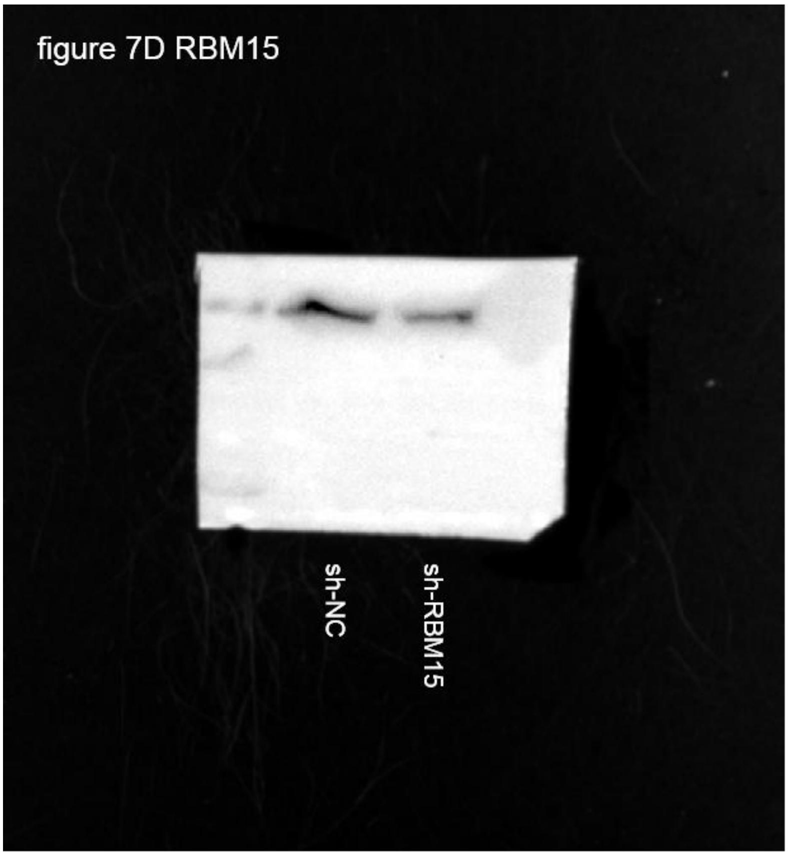

Figure 7D β-actin

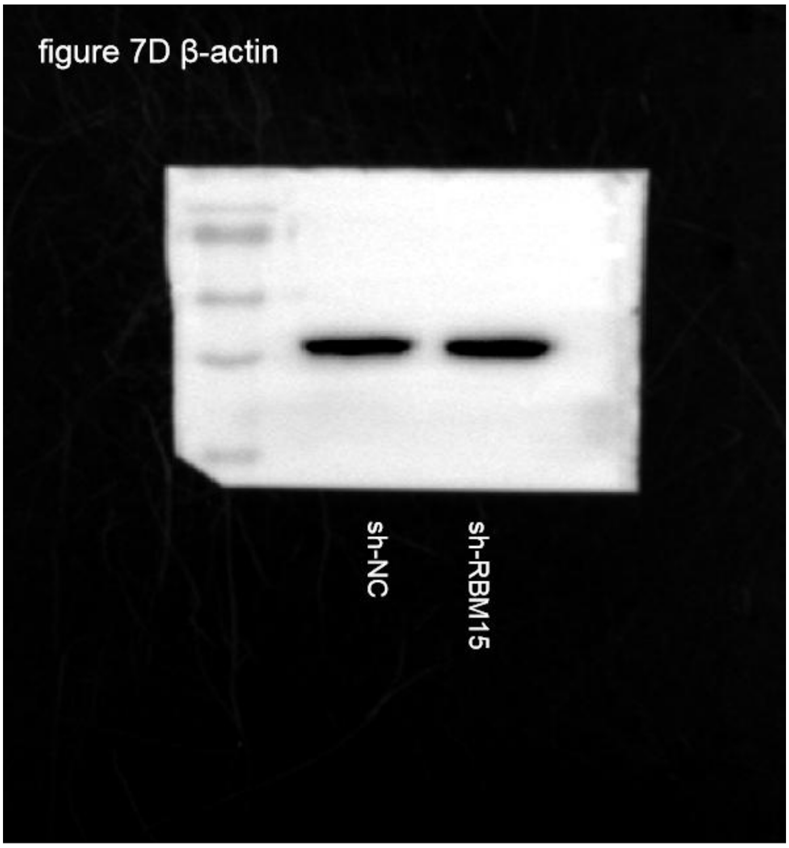

Figure 8C iNOS

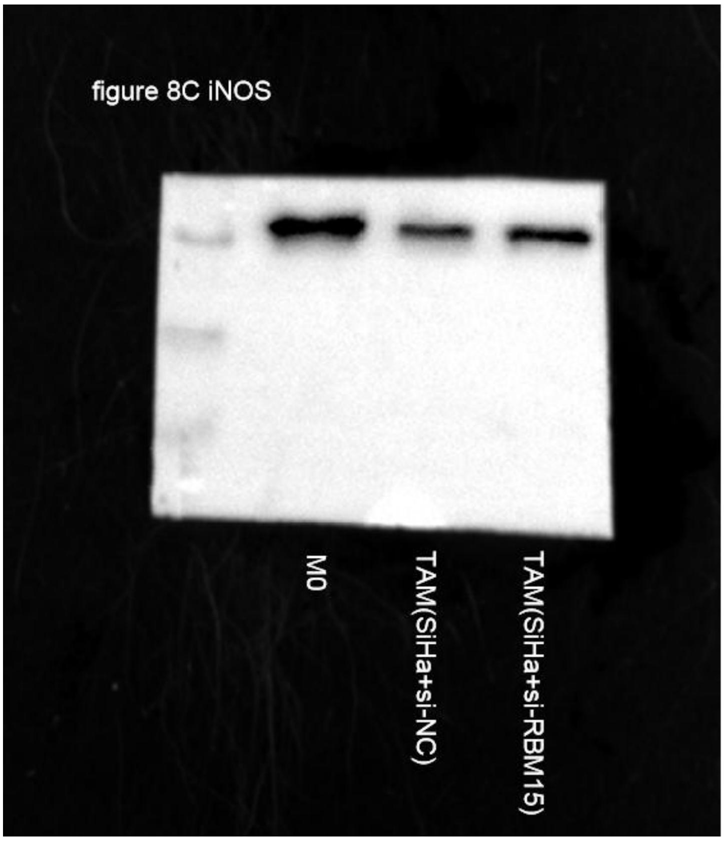

Figure 8C CD206

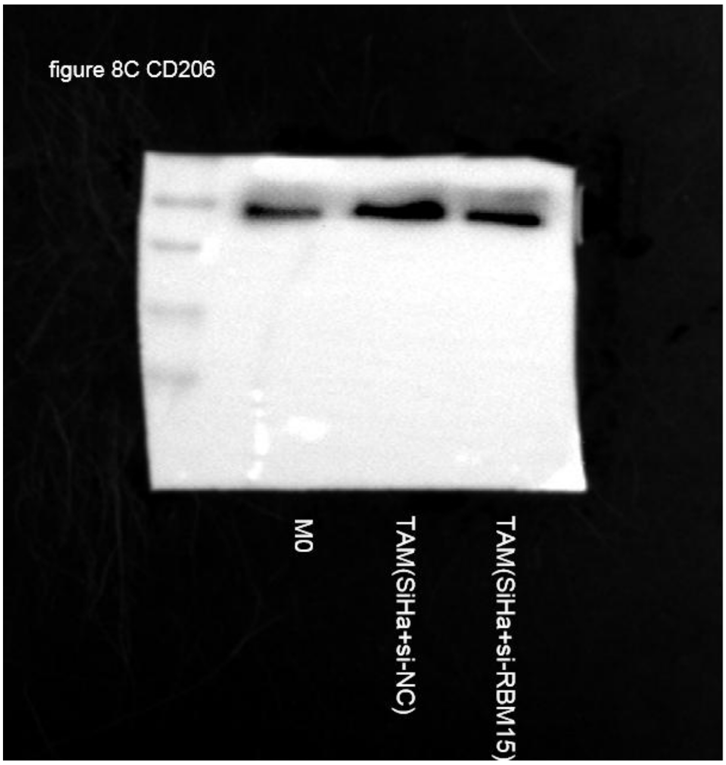

Figure 8C β-actin

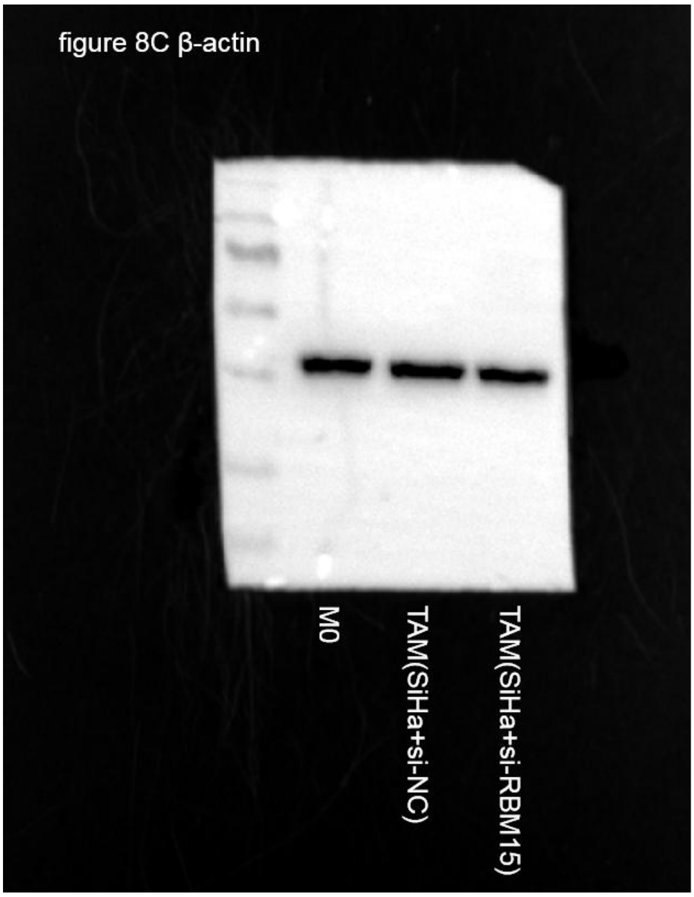

Figure 8D iNOS

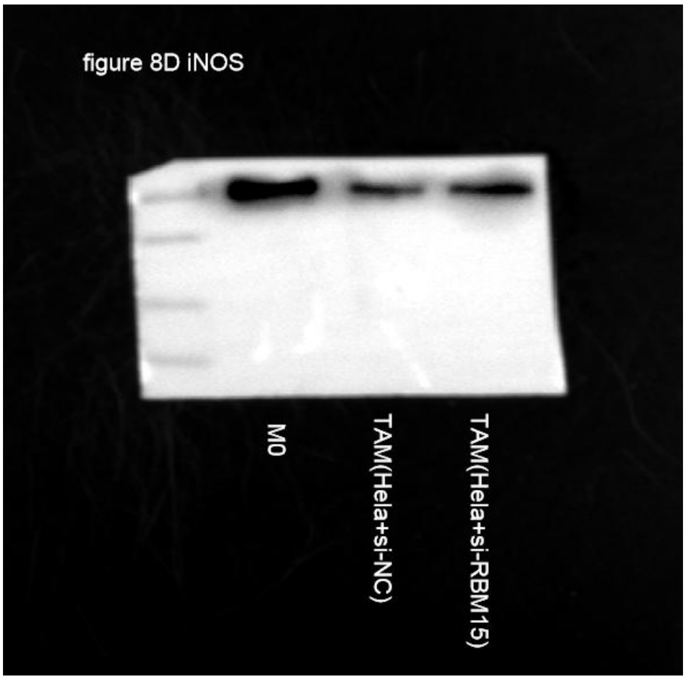

Figure 8D CD206

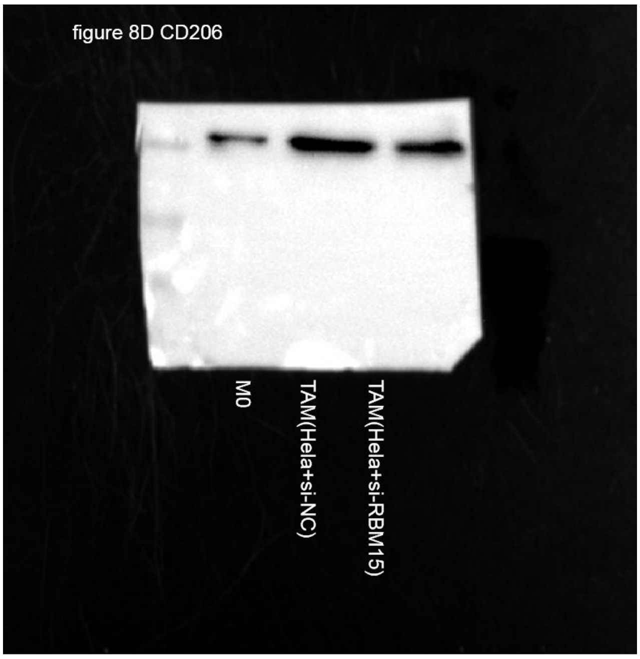

Figure 8D β-actin

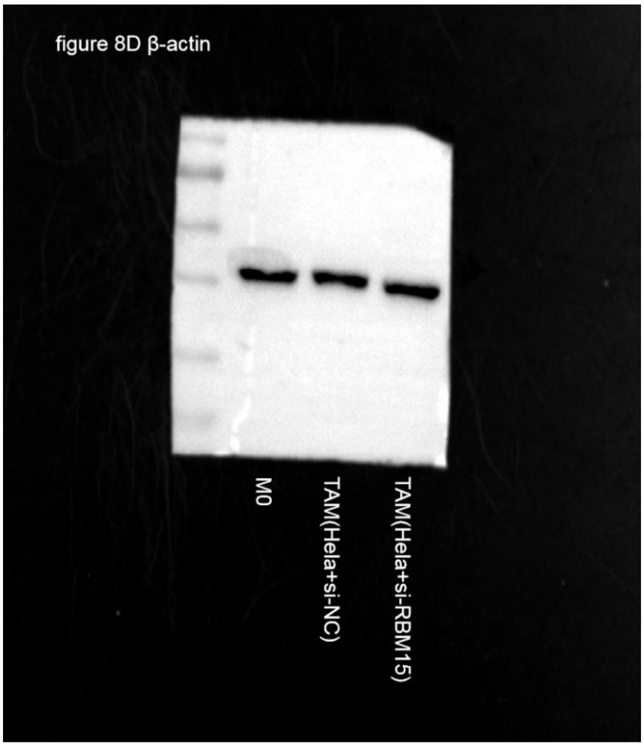

Supplement: Supplementary file 8 — Supplementary Material 8 [file 12885_2023_11163_MOESM8_ESM.pdf]
